# Evaluation of the genomic epidemiology and transmission of *Clostridioides difficile* infection across a community

**DOI:** 10.1017/ash.2022.110

**Published:** 2022-05-16

**Authors:** Brenda Tesini, Samantha Taffner, Trupti Hatwar, Steven Gill, Ghinwa Dumyati, Nicole Pecora

## Abstract

**Background:** *Clostridioides difficile* infection (CDI) is a major cause of morbidity and healthcare costs in the United States. The epidemiology of CDI has recently shifted, with healthcare-associated (HCA) CDI trending downward and community-associated (CA)-CDI becoming more prominent. The cause of this shift is not well understood but may be related to changing genomic epidemiology. We assessed *C. difficile* strains across a CDC Emerging Infections Program (EIP) site in Western New York, including strains from both HCA-CDI and CA-CDI cases to characterize predominating strains and putative transmission across epidemiological classifications and between index and recurrent cases. **Methods:** In total, 535 isolates of *C. difficile* were collected over a 6-month period in 2018 from the Monroe Country, New York, EIP site and were analyzed using whole-genome sequencing (WGS). Standard epidemiological definitions were used to classify cases as hospital onset (HO-CDI); community associated (CA-CDI); community onset, healthcare associated (CO-HCFA-CDI); or long-term care onset (LTCO-CDI). Recurrent cases were defined as those diagnosed within 8 weeks of an initial positive test. Multilocus sequence types (MLSTs) were assigned according to PUBMLST and single-nucleotide polymorphisms (SNPs) were determined using a modified CFSAN analytical pipeline. Cases resulting from putative transmission were defined as those separated by 0–1 core SNPs. **Results:** Of 535 isolates, 454 were from index and 81 were from recurrent cases. The index cases were comprised of CA-CDI (47.4%), CO-HCFA-CDI (24%), LTCO-CDI (8.1%), and HO-CDI (19.3%). Cases with recurrent disease mirrored the epidemiological distribution of the larger set. Common MLSTs included ST2 (12.3%), ST8 (10.5%), ST42 (7.9%), ST58 (4.9%), ST43 (4.5%), and ST11 (4.3%). The previously widespread epidemic strain, NAP1/ST1/RT027 accounted for **Conclusions:** The genomic epidemiology of *C. difficile* across this large community cohort demonstrated a diverse group of strain types that was similarly distributed across epidemiological classifications and between index and recurrent cases. SNP analysis indicated that direct transmission between cases was uncommon.

**Funding:** None

**Disclosures:** None

